# Application of the Performance Index for Monitoring Anaerobic Endurance of Competitive Swimmers

**DOI:** 10.5114/jhk/196823

**Published:** 2025-07-21

**Authors:** Szymon Kuliś, Tomasz Jabłoński, Carlo Rossi, Maciej Skorulski, Anna Kossek, Edyta Sienkiewicz-Dianzenza, Przemysław Pietraszewski, Artur Gołaś, Jan Gajewski

**Affiliations:** 1 Department of Rehabilitation, Józef Piłsudski University of Physical Education, Warsaw, Poland.; 2 Department of Biological Foundations of Physical Education, Institute of Physical Education, Kazimierz Wielki University, Bydgoszcz, Poland.; 3 Sport and Exercise Sciences Research Unit, Department of Psychology, Educational Science and Human Movement, University of Palermo, Palermo, Italy.; 4 Faculty of Physical Education, Józef Piłsudski University of Physical Education, Warsaw, Poland.; 5 Department of Sports Theory and Practice, Institute of Sport Sciences, The Jerzy Kukuczka Academy of Physical Education, Katowice, Poland.

**Keywords:** intensity, interval, swimmers, sports level, endurance

## Abstract

The purpose of the study was to investigate the effectiveness of the performance index (PI) in monitoring anaerobic endurance of adult competitive swimmers during a nine-week intervention. The study included 30 male competitive swimmers. Participants were allocated to an advanced or an intermediate group, taking into account their training experience and sport results. Each participant was tasked with swimming eight lengths of a 25-m pool at maximum speed and full commitment with front crawl and with 15-s rest intervals between subsequent laps at the start of the intervention (pre-test) and nine weeks later (post-test). The performance index determined by the average speeds in successive laps was analysed. No statistically significant differences were observed between the groups in the calculated performance index at the end of the training cycle. A repeated measures ANOVA revealed an interaction between TIME (a repeated factor) and GROUP (a fix factor) (F1.28 = 25.45, p < 0.0001, η^2^ = 0.476). Swimmers from the intermediate group significantly improved their PI (p = 0.0002), while the advanced swimmers did not. Coaches could apply the methodology presented in this study to the specific requirements of their disciplines. The adaptability of the performance index method makes it a valuable tool for assessing anaerobic endurance among athletes of varying experience levels, though it does not serve as a means to directly enhance anaerobic endurance.

## Introduction

Continuous refinement of methodologies for training cycle management and the improvement of anaerobic endurance remain a perpetual pursuit within sports science. Scholarly discourse underscores the imperative for novel assessment techniques that are simple in application and cost-effective for both coaches and athletes (Rago et al., 2022; Zacca and Souza Castro, 2012). Analysing the aerobic and anaerobic maximal values of swimmers throughout a training period can offer valuable insights into their performance, progress, and overall physiological adaptation to the demands of their sport (Scott et al., 2023).

Understanding how these variables change over time could help coaches tailor training programs more effectively for particular swimmers (Campos et al., 2017; Ruiz-Navarro et al., 2024; Thornton et al., 2019). Training monitoring is crucial for effectively managing the sports training process (Strzała et al., 2021). Utilising tools and methods that do not require access to specialised equipment or laboratories can be particularly valuable in this regard. In the literature, various established techniques for assessing and managing athletes’ endurance exist (Bielec et al., 2013; Karakoç et al., 2012; Strzała et al., 2007). However, numerous methods rely on specialised equipment that may not be accessible for financial reasons, particularly in smaller sports clubs or public schools. One method found in the literature to express anaerobic endurance is the performance index (Kuliś et al., 2020; Sienkiewicz-Dianzenza et al., 2009; Stupnicki and Sienkiewicz-Dianzenza, 2004). Stachowicz et al. (2011) introduced the performance index (PI) formula, PI = V_av_/V_max_, where V_av_ represents the mean swimming velocity, and V_max_ denotes the maximum recorded swimming velocity.

The performance index gauges a swimmer’s ability to sustain a maximum velocity throughout consecutive swimming laps, serving as an indicator of their anaerobic endurance. Elevated PI values correlate with superior anaerobic endurance. While Stachowicz et al. (2011) pioneered the application of the performance index in swimming, their study exclusively involved 12-year-old swimmers. Those authors concluded that the performance index, as a measure of anaerobic endurance, might serve as a useful tool in assessing performance changes in youth swimmers. Numerous investigations provide valuable insights into the application of the performance index for assessing anaerobic endurance in other sports disciplines, such as dancing and football (Kuliś et al., 2020; Stacjowicz aet al., 2011).

This study aimed not only to fill the gap on anaerobic capacity of youth swimmers, but also to provide practical tools for coaches and athletes. The use of the PI in daily practice can lead to better monitoring training efficiency. Understanding the relevance between the PI and anaerobic endurance in adult swimmers can offer valuable insights for coaches and swimmers to customise training methods and enhance overall performance. Therefore, the primary objective of the study was to investigate the effectiveness of the PI in monitoring anaerobic endurance of competitive swimmers during a nine-week intervention.

## Methods

### Participants

The study included 30 male competitive swimmers. The swimmers were engaged in competitive swimming for at least five years, and trained five days a week in the training mode set by the coach from their sport club. Some of the swimmers taking part in the experiment were Polish champions in their age category. The study group was divided into an advanced and an intermediate group, based on their training experience, best times at the 50-m sprint and the sport results. According to the classification of McKay et al. (2022), the advanced group was equivalent to the highly trained national level and the intermediate group to the trained developmental. The advanced group consisted of 15 athletes (n = 15) with at least 10 years of training experience, while the intermediate group comprised swimmers (n = 15) with less than 10 years of training experience. In order to qualify for the advanced group, swimmers had to achieve at least 26.5 s at the 50-m swim sprint performed with a front crawl and a minimum of 500 World Aquatics points scored in swimming competitions confirmed at https://www.swimrankings.net/. The study was granted ethical approval from the Ethics Committee of the Józef Piłsudski University of Physical Education, Warsaw, Poland (approval code: SKE 01-31/2023; approval date: 31 January 2023). The research was conducted in accordance with the principles specified in the Declaration of Helsinki. Participants provided their consent in writing after being informed about the purpose, procedures, and benefits of the study. They were also informed that they could withdraw their consent at any time for any reason. The age, body height, body mass and the BMI of swimmers are presented in Table 1.

**Table 1:** Age, body mass, training experience and the BMI of advanced and intermediate group swimmers.

Variable	Intermediate group (n = 15)	Advanced group (n = 15)
**Age** [years]	20 ± 2	24 ± 3.1
**Body height** [cm]	182.3 ± 4.4	184.8 ± 4.5
**Body mass** [kg]	79.7 ± 9.2	83.5 ± 10.5
**BMI**	23.9 ± 2.1	24.4 ± 2.4
**Training experience** [years]	6.9 ± 1.4	11.9 ± 1.5*

* p < 0.05: different than in the intermediate-level group

### Measures

The swimming times were measured in a 25-m pool, with each participant required to swim 8 repetitions of 25-m all-out swimming in front crawl, with 15-s rest intervals between subsequent laps. Athletes started swimming on command, without diving into the water and they took off from the push-off from the wall. Their times were measured from this point using a Finis hand stopwatch after the ready-to-start command. The times were measured by a licensed swimming coach. Each swimmer was tested individually. Before timing their performance in the water, swimmers completed a 15-min general warm-up on land, supervised by their coach. Then, they swam a total of 30 lengths of the pool at a moderate pace tailored to their individual needs to complete the warm-up. The intermediate and advanced swimmers’ times were measured twice (pre-test and post-test), before and after a 9-week intervention during which the swimmers performed five training sessions per week. Additionally, a decrement score was calculated (Oliver, 2009). Table 2 provides a detailed description of the training volume of particular training micro cycles.

**Table 2:** Detailed description of the training volume of particular microcycles.

Microcycle	Training Program
The first and second microcycles (developmental)	**Monday**: Anaerobic Capacity, volume 2 km (main set: 200 m) **Tuesday**: Aerobic Capacity, volume 2.5–3 km (main set: 1.5 km) **Wednesday**: Recovery, volume 2–2.5 km (technique) **Thursday**: Anaerobic Capacity, volume 2 km (main set: 200 m) **Friday**: Aerobic Capacity, volume 2.5–3 km (main set: 1.5 km)
The third microcycle (recovery)	**Monday**: Recovery, volume 1.5–2 km (technique) **Tuesday**: Aerobic Capacity, volume 2-2.5 km (main set: 1 km) **Wednesday**: Recovery, volume 2–2.5 km (technique) **Thursday**: Anaerobic Capacity, volume 2 km (main set: 200 m) **Friday**: Aerobic Capacity, volume 2.5–3 km (main set: 1 km)
The fourth and fifth microcycles (developmental)	**Monday**: Anaerobic Capacity, volume 2 km (main set: 200 m) **Tuesday**: Aerobic Capacity, volume 2.5–3 km (main set: 1.75 km) **Wednesday**: Recovery, volume 2–2.5 km (technique) **Thursday**: Anaerobic Capacity, volume 2 km (main set: 200 m) **Friday**: Aerobic Capacity, volume 2.5–3 km (main set: 1.75 km)
The sixth microcycle (recovery)	**Monday**: Recovery, volume 1.5–2 km (technique) **Tuesday**: Aerobic Capacity, volume 2–2.5 km (main set: 1 km) **Wednesday**: Recovery, volume 2–2.5 km (technique) **Thursday**: Anaerobic Capacity, volume 2 km (main set: 200 m) **Friday**: Aerobic Capacity, volume 2.5–3 km (main set: 1 km)
The seventh and eighth microcycles (developmental)	**Monday**: Anaerobic Capacity, volume 2 km (main set: 200 m) **Tuesday**: Aerobic Capacity, volume 2.5–3 km (main set: 2 km) **Wednesday**: Recovery, volume 2–2.5 km (technique) **Thursday**: Anaerobic Capacity, volume 2 km (main set: 200 m) **Friday**: Aerobic Capacity, volume 2.5–3 km (main set: 2 km)
The final ninth microcycle (recovery)	**Monday**: Recovery, volume 1.5–2 km (technique) **Tuesday**: Aerobic Capacity, volume 2–2.5 km (main set: 1 km) **Wednesday**: Recovery, volume 2–2.5 km (technique) **Thursday**: Anaerobic Capacity, volume 2 km (main set: 200 m) **Friday**: Aerobic Capacity, volume 2.5–3 km (main set: 1 km)

During one training session, participants mainly performed high-volume, low-intensity exercises with short rest intervals in between. Additionally, these exercises were complemented with short duration high-intensity activities. The volume of the set was up to 2,800 m, with short intense workouts taking up 2/3 of the total set according to recommendations of Olbrecht (2015). Based on the obtained times and lengths of the pool, the velocities in metres per second of each lap were calculated. It was decided to do so because the distribution of the obtained velocity values met the assumption of a normal distribution, while it was not always the case for the values of times. Another assumption of the chosen method of calculation was that the values obtained, such as force, speed or power, should be the maximum values and not the minimum values as it was for time. The performance index (PI) assessed the ability to maintain maximum velocity and was calculated using the formula:


$$PI=\frac{Vmax\cdot n}{\sum_{i=1}^{n}V_{i}}$$

where V_max_ is maximal velocity, V_i_ is velocity at each distance, and n is number of distances.

### Statistical Analysis

The normality of the data distribution was assessed using the Shapiro-Wilk test. The results indicated that the data followed a normal distribution. An analysis of variance for repeated measures was conducted to compare velocities, taking a group as a fixed factor (GROUP: Intermediate, Advanced), and time (TIME: pre- test, post-test) along with a lap (LAP: 1 to 8) as repeated factors. PIs were compared using ANOVA involving GROUP and TIME. The Levene’s test was utilised in order to assess the homogeneity of variance within the sample. The Tukey test was used for post-hoc comparisons. A paired *t*-test for independent groups was applied to determine differences in age, body mass, and training experience. Pearson correlation analyses were used to examine the relationship between the decrement score and the PI. The results were analysed using Jamovi version 2.3.21 software, with the significance level set at α = 0.05.

## Results

**Figure 1:**
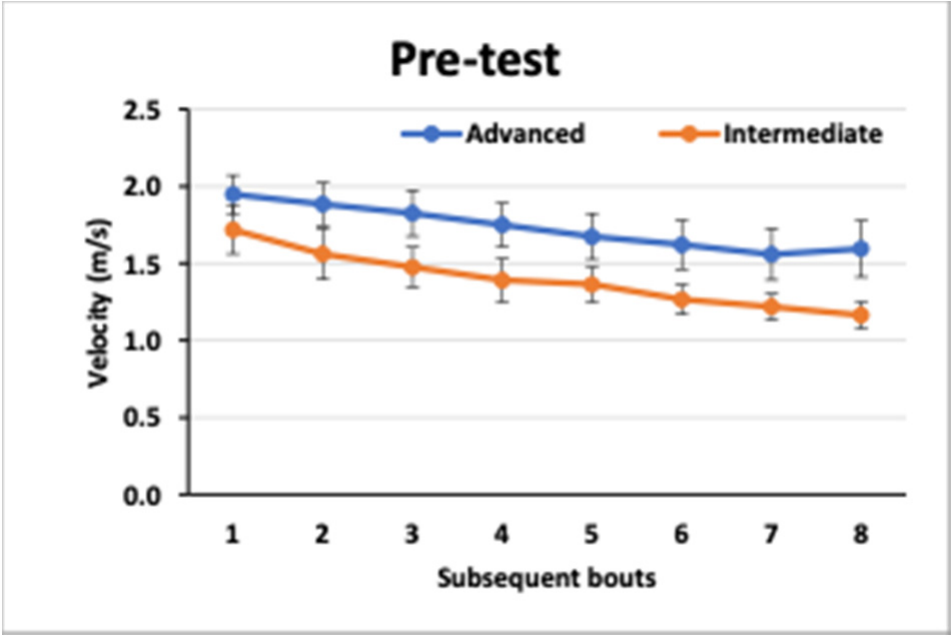
The mean velocities (± SE) obtained by intermediate and advanced swimmers in the pre-test in each lap.

**Figure 2:**
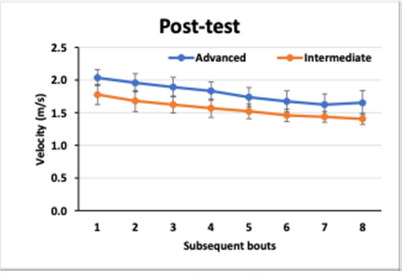
The mean velocities (± SE) obtained by intermediate and advanced swimmers in the post-test in each lap.

The mean velocities (± SE) obtained by intermediate and advanced swimmers in pre-test and post-test in each lap are presented in Figures 1 and 2, respectively.

The analysis of variance revealed a number of differences between the factors analysed and the velocities. As shown in Figures 1 and 2, the two groups presented differences in their swimming velocities (GROUP: F_1.28_ = 38.87, *p* < 0.0001, η^2^ = 0.562). Both groups enhanced their performance considering swimming velocity (TIME: F_1.28_ = 31.2, *p* = 0001, η^2^ = 0.122), yet the increase varied between the two groups (GROUP x TIME: F_1.28_ = 14.99, *p* < 0001, η^2^ = 0.349). The mean velocities for successive laps decreased in both groups (LAP: F_7.196_ = 187.07, *p* < 0.0001, η^2^ = 0.864), and this trend did not differ between the two groups (GROUP x LAP: F_7.196_ = 1.90, *p =* 0.0708, η^2^ = 0.064). Furthermore, the decrease in velocity over successive laps significantly decreased in both groups after the intervention (TIME x LAP: F_7.196_ = 2.18, *p =* 0.0374, η^2^ = 0.072), however, to a different extent (F _7.196_ = 4.04, *p =* 0.0004, η^2^ = 0.126).

The values of the PI are presented in Table 2. Considering that the PI is associated with a decrease in velocity, it therefore synthetically describes anaerobic endurance. It appeared that PI values changed differently in both groups during the intervention, as evidenced by the significant interaction between the GROUP and TIME factors (F_1.28_ = 25.45, *p* < 0001, η^2^ = 0.476).

The post-hoc tests revealed that PI values significantly changed from the pre-test to the post-test for the intermediate swimmers (*p =* 0.0002), but not for the advanced ones (*p =* 0.8834). The appropriate allocation to groups was also confirmed by the obtained PI value at the very beginning of the intervention. This was confirmed by the significant difference found in the PI before the intervention between the intermediate and advanced groups (*p =* 0.0003). After the intervention, no significant improvement in the PI was detected in the advanced group (*p =* 0.8661). Analysis of variance revealed that both mean and maximum velocity increased significantly following the intervention (F_1,28_ = 87.15, *p* < 0001, η^2^ = 0.757 and F_1,28_ = 80.37, *p* < 0001, η^2^ = 0.74, respectively). Additionally, significant interaction between group and repetition occurred for average velocity and the intermediate group achieved greater improvement than the advanced group (F_1,28_ = 14.99, *p =* 0006, η^2^ = 0.349). The average values (± SD) related to endurance recorded in the study group are presented in Table 3.

**Table 3:** Mean (± SD) values of the obtained times, velocity’s and performance indexes in the intermediate and advanced groups at the beginning (pre-test) and the end of the intervention (post-test).

Variable	Intermediate (n = 15)		Advanced (n = 15)	
	Pre-test	Post-test	Pre-test	Post-test
**Lap 1 [s]**	14.7 ± 1.5	14.2 ± 1.4	12.9 0.9	12.3 ± 0.9
**Lap 2 [s]**	16.2 ± 1.8	15.0 ± 1.2	13.3 ± 1.1	12.8 ± 1
**Lap 3 [s]**	17.1 ± 1.7	15.5 ± 1.3	13.8 ± 1.2	13.3 ± 1.2
**Lap 4 [s]**	18.1 ± 1.9	16.1 ± 1.9	14.4 ± 1.2	13.7 ± 1.2
**Lap 5 [s]**	18.4 ± 1.5	16.6 ± 1.8	15.1 ± 1.4	14.5 ± 1.5
**Lap 6 [s]**	19.9 ± 1.6	17.4 ± 2.4	15.6 ± 1.7	15.1 ± 1.9
**Lap 7 [s]**	20.6 ± 1.4	17.7 ± 2.7	16.2 ± 1.8	15.6 ± 1.9
**Lap 8 [s]**	21.6 ± 1.6	18.10 ± 2.3	15.9 ± 1.9	15.4 ± 2.1
**Mean V [m/s]**	1.39 ± 0.1	1.56 ± 0.1**^###^**	1.73 ± 0.1^***^	1.79 ± 0.2^##^^***^
**Max V [m/s]**	1.72 ± 0.17	1.79 ± 0.2**^###^**	1.95 ± 0.1^***^	2.02 ± 0.1^###^^***^
**PI**	0.81 ± 0.1	0.87 ± 0.1**^###^**	0.89 ± 0.04^***^	0.88 ± 0.1

*** different compare to the intermediate group at p < 0.001, **^###^** different than in the pre-test at p <0.001, **^##^** different than in the pre-test at p < 0.01

The average decrement score in advanced swimmers before the intervention was 13.60% ± 5.35%, and it was 14.60% ± 6.16% after the intervention. The correlation before the intervention between the decrement score and the PI was R = −0.98 and after the intervention it was R = −0.99. Among the swimmers in the intermediate group, the average decrement score before the intervention was 25.13% ± 4.66% and it was 16.63% ± 8.16% post-intervention. The correlation between the decrement score and the PI was R = −0.97 pre-interventions and R = –0.99 post-intervention.

## Discussion

The main findings of the study indicate the effectiveness of the performance index (PI) method in assessing and distinguishing among athletes with different levels of training experience based on their anaerobic endurance. The notable interaction in the analysis of variance observed between the intermediate and advanced groups following the intervention underscores the dynamic nature of anaerobic endurance and the utility of the PI for monitoring the training processes. Furthermore, the post hoc tests revealed a significant change in the PI value from the pre-test to the post-test in the intermediate group, emphasising the potential for improvement in anaerobic endurance through targeted training methods. The advantage of the PI is that it describes the ability to maintain maximum speed over the entire distance. There is no assumption that the speed drop is linear, as it correlates with the relative decrement.

The significant increase in PI values in the intermediate group can be attributed to the large increase in average velocity. This indicates an increase in endurance of the athletes. In the advanced group, no increase in the PI was recorded, which was related to a proportional increase in both maximum and average velocity. The observed increase in PI values for the intermediate swimmers suggests the adaptability and potential for enhancement of anaerobic endurance in swimmers with less than 10 years of training experience and indicates the usefulness of the method used in the study. Other researchers have also drawn similar conclusions (Sienkiewicz-Dianzenza et al., 2009). Both the decrement score and PI indicators exhibit a strong correlation. However, the PI specifically quantifies the ability to maintain speed by expressing the average speed as a percentage of the maximum speed across a series of lengths. In contrast, the decrement score measures the percentage by which the average time exceeds the minimum time. Essentially, both indicators are equivalent in their purpose. However, the PI was formally developed and published earlier than the decrement score (Stupnicki and Sienkiewicz-Dianzenza, 2004). According to Stachowicz et al. (2011), the PI can be a useful tool for comparing anaerobic endurance between males and females, as well as between different exercise tests consisting of repeated, short, maximal efforts. This implies that the PI can be beneficial in assessing and monitoring anaerobic performance in untrained individuals (Tomczak and Stupnicki, 2014). There has already been work in the literature dealing with the impact of critical speed over shorter distances (Mitchell et al., 2018, 2019). However, our research represents a pioneering effort to identify significant differences between swimmers of different sports levels.

Furthermore, in our study, the time between the two measurements (pre- vs. post-intervention) was rather short, especially considering other studies, where the longest time between the two measurements was six months (Tomczak and Stupnicki, 2014). Another difference concerns the study participants as in other studies, the study groups did not differ statistically in terms of the PI values (Stachowicz et al., 2011). Notably, the lack of a significant improvement with relatively small effect size in the PI of the advanced group after the intervention raises questions about the potential application of this method with regard to anaerobic endurance in swimmers with extensive training experience. However, the lack of significant rate progress in the advanced group may be due to the already high level of anaerobic endurance at the beginning of the study in the pre-test. It is conceivable that the identical training program implemented in both the intermediate and advanced groups may not be adequate to drive progress in the advanced group relative to the intermediate group. This phenomenon could provide useful information for coaches and underscores the need for specific and tailored training programs to further improve anaerobic endurance in the group of advanced swimmers. The advantage of the chosen research method is, however, its extensive implementation in various sports disciplines (Kuliś et al., 2020). Other authors have also recommended focusing on measuring and adjusting training programs using in-water testing, as it closely resembles the movement of actual swimming and could provide direct feedback to swimmers and coaches (Madureira et al., 2012; Strzała et al., 2021). The presented method could be equivalent to methods in which oxygen measurements are used (Rębiś et al., 2022).

When interpreting our findings, it is imperative to acknowledge the inherent individual variability in athletes’ responses to training stimuli. Factors such as genetic predispositions and inherent physiological differences could contribute to the diverse outcomes observed in the anaerobic endurance improvements. It is worth noting that participants in the present study covered fairly short distances in front crawl. It is evident that the distances and styles vary at sports competitions (Ponimasov and Bolotin, 2019). Considering this aspect, swimmers often adopt a range of strategies for balancing their strength and endurance, particularly when tackling longer distances (Campos et al., 2017; Ponimasov and Bolotin, 2019; Veiga et al., 2019). There might be a valid concern about the suitability of the PI for use over longer distances. It seems that its greatest usefulness may be found in measuring anaerobic endurance during distances of 50, 100 and 200 m, where athletes attempt to cover each length at maximum velocity (Aspenes et al., 2009; Bielec et al., 2013).

The role of psychological factors in anaerobic performance also merits consideration. Motivation levels, mental resilience, and adherence to training programs could significantly impact the observed changes in the PI during tests. Moreover, to comprehensively evaluate the utility of the PI, a comparative analysis with other established assessment methods, such as lactate threshold testing or critical swim velocity (Fernandes et al., 2010; Mavroudi et al., 2023; Meckel et al., 2012) could offer a more nuanced understanding of its strengths and potential areas for improvement. Many sports clubs lack the necessary specialised tools to monitor their athletes’ endurance and track progress in recreational or competitive swimming. The PI calculation method applied in this study can have practical implications for physical education teachers, swimming instructors, swimmers, and other related professionals. They could use the method presented in here as a framework and then tailor it to better fit their specific needs and goals.

In conclusion, the results of this study demonstrate the effectiveness of the PI method in assessing and distinguishing among athletes with varying levels of training experience based on their anaerobic endurance. The significant interaction observed between the intermediate and advanced groups following the nine-week intervention highlights the potential for improvement in anaerobic endurance through targeted training methods, especially for swimmers with less than 10 years of training experience.

## Conclusions

Understanding the relative strengths and limitations of different assessment tools will contribute to a more comprehensive approach to evaluating anaerobic endurance. The versatility of the PI method suggests its potential application beyond swimming. Coaches and trainers across various sports could explore its use, adapting the methodology presented in this study to align with the specific demands of their respective disciplines. In practical terms, coaches, physical education teachers, swimming instructors, and related professionals can leverage the insights from this study to refine their training approaches. The adaptability of the PI method and its potential to inform individualised training programs make it a valuable tool for enhancing anaerobic endurance among swimmers with varying levels of experience.
